# Modeling and Synthesis of Alumina Whiskers Based on the Vapor Deposition Process

**DOI:** 10.3390/ma10101192

**Published:** 2017-10-17

**Authors:** Wei Gong, Xiang-Cheng Li, Bo-Quan Zhu

**Affiliations:** 1 Glarun Technology Co. Ltd., Nanjing 211106, China; gongwei1938@163.com; 2 The State Key Laboratory of Refractories and Metallurgy, Wuhan University of Science and Technology, Wuhan 430081, China

**Keywords:** growth simulation, generation mechanism, Al_2_O_3_ whiskers, surface energy

## Abstract

This study simulated the bulk structure and surface energy of Al_2_O_3_ based on the density of states (DOS) and studied the synthesis and microstructure of one-dimensional Al_2_O_3_ whiskers. The simulation results indicate that the (001) surface has a higher surface energy than the others. The growth mechanism of Al_2_O_3_ whiskers follows vapor–solid (VS) growth. For the (001) surface with the higher surface energy, the driving force of crystal growth would be more intense in the (001) plane, and the alumina crystal would tend to grow preferentially along the direction of the (001) plane from the tip of the crystal. The Al_2_O_3_ grows to the shape of whisker with [001] orientation, which is proved both through modeling and experimentation.

## 1. Introduction

Compared with fibers with diameters in the millimeter or micrometer scale, Al_2_O_3_ whiskers with a sub-micrometer scale have the better advantages of high melting point, high specific strength, high specific modulus, high temperature oxidation resistance, and high chemical compatibility [1,2]. All these features give rise to the application of whiskers in the composite as a kind of excellent reinforcement phase in the fields of biology, aerospace, mechanics, polymers, and so on [3,4]. For example, D. Gomez-Garcia et al. [1] reported that ceramics with 3 wt. % Al_2_O_3_ whiskers exhibit comparable strength to pure ceramics, and the fracture toughness could be increased from 4.2 MPa·m^1/2^ to 5.6 MPa·m^1/2^. S. Gonzalez-Lopez et al. [5] found that the addition of about 3 wt. % Al_2_O_3_ whiskers would double the flexural strength of alumina ceramics and benefit their application in biological fields. A. Ares et al. [6] determined that only 5 wt. % Al_2_O_3_ whiskers filler could increase the thermal stability of a polyethylene matrix by around 37% and possess excellent compatibility with the matrix. J. Corrochano et al. [7] added 10 vol. % Al_2_O_3_ whiskers in an aluminum matrix and enhanced the strength by five or six times. M.A. bdullah et al. [8] prepared Al_2_O_3_ whisker-reinforced zirconia ceramics and found that the rupture strength could reach 1325 MPa when 10 wt. % whiskers were introduced. At the same time, the Vickers hardness could be increased to 13.8 GPa. The above research work shows that Al_2_O_3_ whiskers definitely play a key role in ensuring excellent performance for composites or ceramics industries. 

With the development of industrial manufacturing and material sciences, more and more researchers are focused on the preparation and application of such oxide whiskers as alumina. In 1957, W.W. Webb and W.D. Forgeng et al. [9] first fabricated alumina whiskers by heating mixtures of alumina and TiAl_3_ at 1300~1450 °C for 2–24 h in a stream of hydrogen. Recently, many efforts have been made to obtain large-yield preparation of alumina whiskers to meet the requirements of industry applications. For example, C.N.R. Rao et al. [3] synthesized Al_2_O_3_ whiskers by heating a mixture of Al and graphite powders in a zirconia boat under flowing Ar at 1300 °C for 6 h, and it grow along the [001] direction with high aspect ratios. E. Mudra et al. [2] used the sintering method to obtain Al_2_O_3_ electrospun fibers that were tens of micrometers in diameter. W.B. Dai et al. [4] obtained transition Al_2_O_3_ whiskers by the thermal decomposition of ammonium hexafluoroaluminate, and the whiskers’ diameter was decreased to the near sub-micrometer scale. Y.H. Cui et al. [10] synthesized single crystal Al_2_O_3_ whiskers by an in situ method and found a screw dislocation growth mechanism followed by alumina whisker growth. The authors’ previous research [11] reported that the synthesis of Al_2_O_3_ whiskers through the carbon-assisted method possessed the characteristics of easy operation and flexible reaction conditions. However, the above research only considers the synthesis of Al_2_O_3_ whiskers, but does not explore the simulation and growth mechanism of these whiskers.

In theory, a detailed and deep understanding of the simulation and modeling of one-dimensional alumina whiskers would play a key role in controlling the growth and manufacture experiments of these materials. Amongst these researching processes, the surface energy could determine their growth direction and the morphology according to crystal growing theory. To date, many researchers have studied the surface energy during the crystal growth process based on density functional theory (DFT) [12,13,14,15], and the corresponding synthesis mechanism of one-dimensional materials could theoretically be interpreted. For instance, Q.J. Liu et al. [16] calculated the surface energy of the (001) crystal face for cubic SrHfO_3_, which indicated that—as the function of terminated slab—SrO was more stable than HfO_2_. G.H. Chen et al. [17] also studied the electronic properties and structure of HfO_2_ surfaces, and found that the (110) and (111) crystal faces could be terminated when a single oxygen layer was the most energetically favorable. X. Li et al. [18] studied the MgAl_2_O_4_ (111) surfaces and found that the O_2_(Mg)- and Al(O)- terminated surfaces were of higher surface energy, which meant that it would easily interact with the other compounds and had thepotential to grow in vacuum. P.L. Liu et al. [19] reported that the Zn-terminated ZnO (0001) polar surface with high surface energy was chemically active with the O_2_ gas phase, leading to the rapid growth of nanotips along the *c*-axis direction. Therefore, the investigation of surface energy based on DFT would probably be successful in revealing the growth process and mechanism of one-dimensional whiskers. 

Based on the above described research, the objective of this paper was to determine the relative surface stability and the preferred orientation Al_2_O_3_ through DFT under thermodynamic equilibrium conditions. Furthermore, the Al_2_O_3_ whiskers synthesis by the carbon-assisted method and its generation mechanism combined with surface energies was studied.

## 2. Simulation and Experimentation

The modeling and calculations in our work were performed with the density function theory (DFT) using plane-wave pseudopotential. The localized density approximation (LDA) with the Ceperley–Alder–Perdew–Zunger (CA–PZ) functional was implemented based on the Cambridge Serial Total Energy Package (CASTEP) code, and the model of Materials Studio software [20]. The ionic cores were represented by ultrasoft pseudo potentials for Al and O atoms, and the valence states of Al and O were denoted as Al *3s*^2^*3p*^1^ and O*2s*^2^*2p*^4^, respectively. The cutoff energy of the place-wave basis was 340 eV throughout the bulk and slab models. The special k-point sampling of the Monhkorst-Pack scheme was used to approximate the Brillouin zone integrations. This set of parameters could assure the total energy convergence of 5.0 × 10^−6^ eV/atom, the maximum force of 0.01 eV/Å, the maximum stress of 0.02 GPa, and the maximum displacement of 5.0 × 10^−4^ Å. In order to make the calculation of Al_2_O_3_ bulk, a mesh size of 6 × 6 × 2 was used for k-point sampling.

Flake graphite (95.00 wt. % with *d*_50_ = 0.088 mm) and metallic Al powders (99.50 wt. %) were used as raw materials. The raw materials were mixed according to the mass ratio of Al:C = 3:7. The mixture was heated at 1300 °C for 3 h under an Ar atmosphere. The phase composition and crystalline of the as-synthesized sample (alumina whiskers) were analyzed by X-ray diffraction (XRD, X’Pert Pro, Philips, Amsterdam, The Netherlands) with a copper anode (Cu-K_α1_, λ = 0.15405 nm) working at 40 kV and 40 mA, scanning in continuous mode at a rate of 2°∙min^−1^. The microstructure and morphology of alumina whiskers were determined by scanning electron microscopy (FESEM, Nova 400 Nano SEM, FEI, Cambridge, UK) supported by energy-dispersive X-ray spectroscopy (EDS, INCA, IE350 penta FET X-3, Oxford, UK). The high resolution and crystalline structure were measured by transmission electron microscopy (TEM, JEM-2100 UHR, JEOL, Tokyo, Japan) and energy dispersive spectroscopy (EDS, Phoenix, Oxford, UK). Furthermore, the growth direction of alumina whiskers was depicted by TEM analysis. 

## 3. Results and Discussion

### 3.1. Band Structure and Density of States 

α-Al_2_O_3_ has a corundum structure (space group R $\bar{3}$ c, No.167), which the O atoms stack in ABAB style, and Al atoms occupy the octahedral sites in the space group. The stable phase α-Al_2_O_3_ contains two units of Al_2_O_3_ in a rhombohedral representation. Before the surface calculations, the bulk model of Al_2_O_3_was calculated and compared with experimental values in order to examining the reliability of the model. The calculated and theoretical values of the lattice parameters are listed in Table 1. It can be found that the optimized lattice parameter calculated by LDA is 5.12 Å, which is in good agreement with the experimental result of 5.14 Å [20] and other theoretical calculations [12,13,14]. 

**Figure 1:**
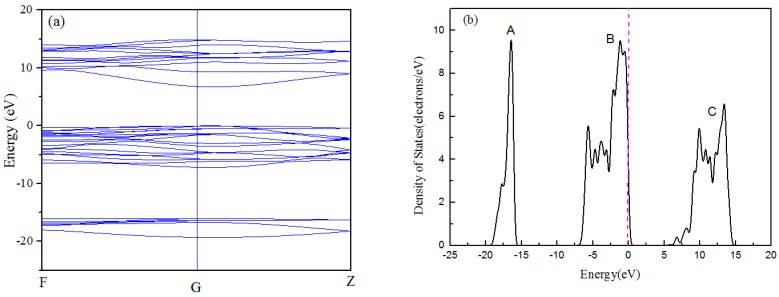

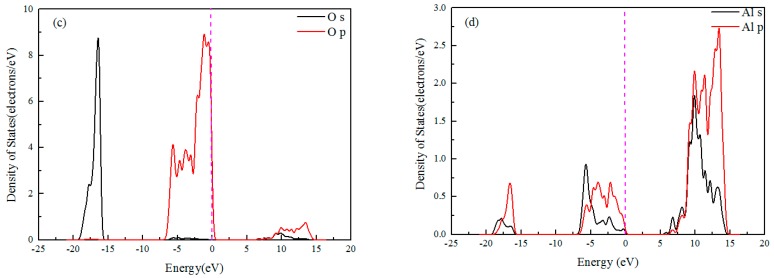
The band structure (**a**); density of states (DOS) (**b**); and projected DOS (**c**,**d**) of Al_2_O_3_.

The band structure and density of states (DOS) of Al_2_O_3_ are shown in Figure 1. From the band structure of Al_2_O_3_ in Figure 1a, it can be seen that the top of the valence band and bottom of the conduction band meet at the G point with a direct band gap of 6.704 eV, which is consistent with the previous theoretical results [21]. However, this value is smaller than its experimental value of 8.8 eV due to the LDA underestimating short-range repulsive interaction. Figure 1b–d show that the valence band of Al_2_O_3_ has three regions. The lower region between −20 eV to −15 eV is attributed to the O *2s* state, and the middle region is composed of O *2p* states hybridized with Al *3s* and *3p* states. The upper region is composed of Al *3s*, Al *3p*, and O *2p* states, while the Al *3s* and Al *3p* states mainly contribute to the conduction band. The above results depict that Al-O bonding in Al_2_O_3_ reveals strong ionic characteristics. This agrees well with previous calculated results of Al_2_O_3_ based on DFT computational methods [22], and proves that the LDA calculations could be used to study the surface of Al_2_O_3_.

### 3.2. Surface Energies Calculation

**Figure 2:**
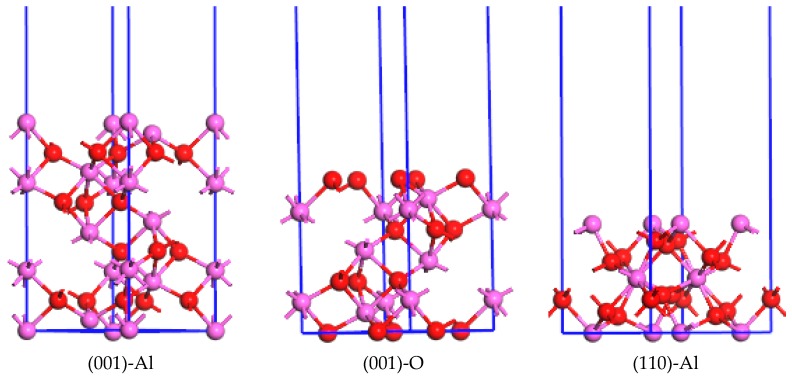

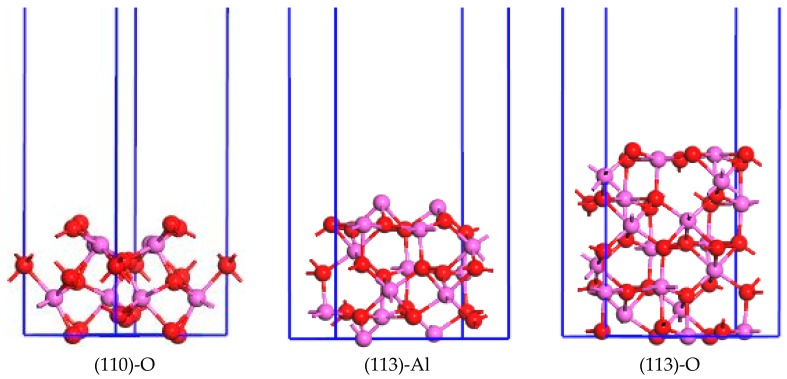
The ball and stick model for the surface structures of Al_2_O_3_. “-Al”and “-O” mean the surfaces terminated by Al atoms and O atoms, respectively. 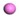 Al atoms, 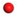 O atoms.

In order to determine the relative surface stability and the preferred orientation of Al_2_O_3_ under thermodynamic equilibrium conditions, the state of the surface energy of the crystals becomes very important. To simulate the surface energies of Al_2_O_3_, the slab model was used and the periodic boundary conditions were applied to the surface super cell under a vacuum region. The modeling of a slab of atomic layers is illustrated in Figure 2. The results showed that the vacuum layer is designed as 15 Å to avoid the interactions among periodic slabs of atomic layers. The unit cells of 1 × 1 are used for the low Miller index (i.e., (001), (110), and (113)) surface of Al_2_O_3_ in our calculations and modeling. For Al_2_O_3_ crystal, the surfaces of (001), (110), and (113) may be terminated and determined by Al or O layer. The atomic layers of 9 or 7 are used in the slab for the surface of Al_2_O_3_ here, as shown in Figure 2. The k-meshes of (001),(110), and (113) surfaces of Al_2_O_3_ in the calculations are 6 × 6 × 1, 4 × 5 × 1, and 3 × 5 × 1.

We take the surface energy (*E^surf^*) to represent the stability of various surfaces, and denote the surface energy as the cut-off energy of the crystal. For Al_2_O_3_, *E^surf^* is calculated as:

(1) Esurf=12A[Etotslab−12NAlEtotAl2O3−(N0−32NAl)μ0]

As explained in Reference [17], the value of $E_{tot}^{slab}$ is the total energy of the surface slab, and $E_{tot}^{Al_{2}O_{3}}$ refers to the energy of bulk Al_2_O_3_ per formula unit. The symbol of A is the surface area of the surface slab. *N_Al_* and *N_O_* are the number of Al atoms and O atoms in the slab, respectively. The excessive oxygen beyond stoichiometric Al_2_O_3_ units in the slab is denoted as N0−32NAl. *μ_O_* varies between $\mu_{0}^{0}$ and $\mu_{0}^{Al_{2}O_{3}}$. As we all know, the thermodynamically allowed chemical potential is key to study the dependence of surface stability on the environment. So, $\mu_{0}^{0}$ is the chemical potential of oxygen and is taken as half of the total energy of one O_2_ molecule. The relation between $\mu_{0}^{Al_{2}O_{3}}$ and $\mu_{Al}^{Al_{2}O_{3}}$ is given by a formula:

$$2\mu_{Al}^{Al_{2}O_{3}}+3\mu_{0}^{Al_{2}O_{3}}=E_{tot}^{AL_{2}O_{3}}$$

The formation energy ($\Delta E_{f}^{Al_{2}O_{3}}$) of bulk Al_2_O_3_ is defined as:

$$\Delta E_{f}^{Al_{2}O_{3}}=E_{tot}^{Al_{2}O_{3}}-2\mu_{Al}^{0}-3\mu_{0}^{0}$$

where $\mu_{Al}^{0}$ is the chemical potential of Al, which is taken as the total energy of bulk Al. The variation range of *μ*_0_ can be obtained by:

13ΔEfAl2O3+μ00≤μ0≤μ00

**Figure 3:**
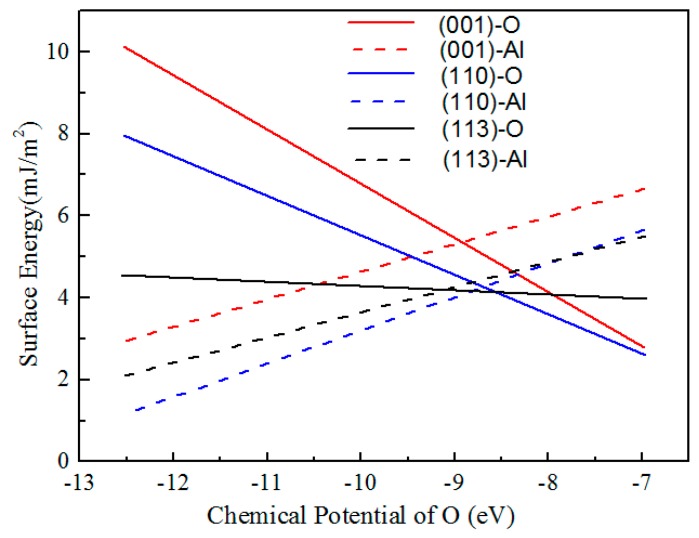
Surface energies for various surface of Al_2_O_3_ versus chemical potential of oxygen.

In order to compare the stability of Al_2_O_3_ surfaces, the calculated surface energies for (001)-O, (001)-Al, (110)-O, (110)-Al, (113)-O, and (113)-Al versus the chemical potential of oxygen are plotted in Figure 3. Under oxygen-rich conditions, the surface energies of low Miller index surfaces of Al_2_O_3_ follow in the sequence of (001)-Al > (110)-Al > (113)-Al > (113)-O > (001)-O > (110)-O. Under oxygen- deficient conditions, the sequence converts to the trend of (001)-O > (110)-O > (113)-O > (001)-Al > (113)-Al > (110)-Al. The result indicates that the (110) surface is the most stable surface of Al_2_O_3_. For the (001) surface with the higher surface energy, the driving force for crystal growth would be more intense in the (001) plane, and the alumina crystal would tend to grow preferentially along the direction of the (001) plane from the tip of the crystal. As a consequence, the Al_2_O_3_ grew to the shape of a whisker with [001] orientation [19].

### 3.3. Phase Composition and Microstructure of the As-Synthesized Sample 

**Figure 4:**
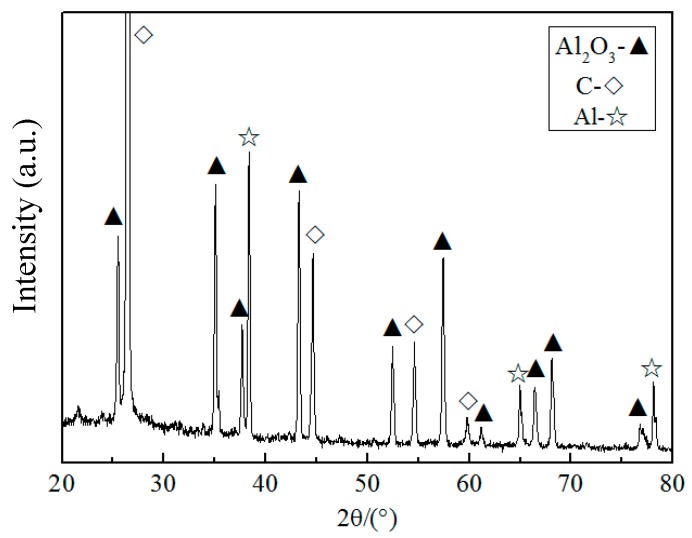
XRD pattern of the as-synthesized sample.

According to the modeling and calculation of one-dimensional Al_2_O_3_ whiskers, detailed preparations were carried out. The XRD pattern of the as-synthesized sample heated at 1300 °C for 6 h is shown in Figure 4. The major phases in the as-synthesized samples are graphite, Al, and Al_2_O_3_. The strongest diffraction peak of graphite is detected at 26.5°. Apart from graphite, the diffraction peaks at 25.49°, 35.05°, 37.68°, 43.25°, 52.48°, and 57.42° can be indexed as the (012), (104), (110), (113), (024), and (116) crystal planes of Al_2_O_3_, respectively. The diffraction peaks of Al_2_O_3_ are the results of the metallic Al powders reacting with the remnant oxygen under high temperatures. This agrees with the authors’ previous research [11].

**Figure 5:**
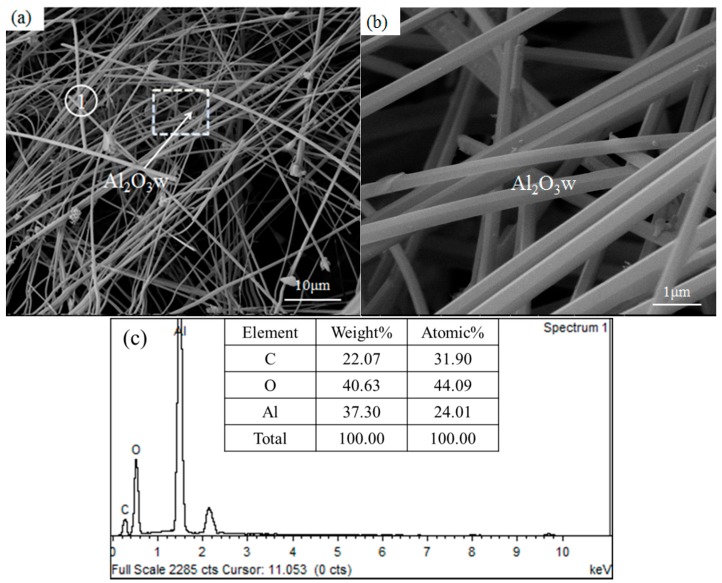
SEM images (**a**,**b**) and EDS spectrum (**c**) of the as-synthesized samples: Al_2_O_3_w means Al_2_O_3_ whiskers.

The SEM images and EDS spectrum of Al_2_O_3_ whiskers are shown in Figure 5. One-dimensional whiskers were formed in the as-synthesized sample, and the whiskers were 50–500 nm in diameter and hundreds of microns in length, as shown in Figure 5a,b. The EDS spectrum (Figure 5c) at point 1 of Figure 5a are composed of three elements: O, Al, and carbon. The EDS spectrum clearly indicates that the whiskers have the composition of Al_2_O_3_. This is in conformity with the Al_2_O_3_ phase determined by the XRD diffraction pattern (Figure 4). The high-magnification SEM images in Figure 5b show that the Al_2_O_3_ whiskers are smooth and flat, belonging to the hexagonal crystal system. There are no catalyst or liquid droplets at the ends of the whiskers, which means that the growth mechanism of Al_2_O_3_ whiskers follows the vapor–solid (VS) mechanism.

**Figure 6:**
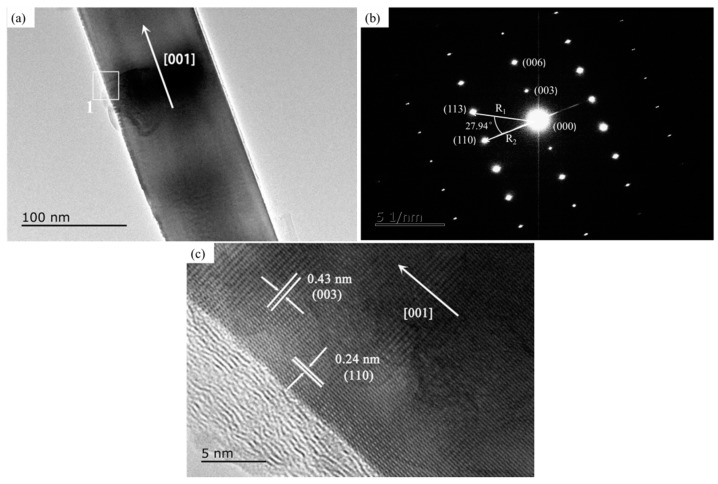
TEM image of Al_2_O_3_ whisker: (**a**) general TEM image; (**b**) SAED of Figure 6a; (**c**) high-resolution TEM.

In order to further explore the morphology of Al_2_O_3_ whiskers, a TEM analysis was conducted. The TEM image, selected area electron diffraction (SAED), and high-resolution TEM image of Al_2_O_3_ whiskers are shown in Figure 6. Figure 6a is the general TEM image of Al_2_O_3_ whiskers, with a diameter of about 100 nm. The SAED of the whiskers is depicted in Figure 6b. It could be found that the whiskers take on a single crystal with a hexagonal structure. According to the SAED, each diffraction spot corresponds to certain crystal face. The interplanar spacing of the two selected crystal faces and the angle (*θ*) of the two selected crystal faces were measured, respectively, as shown in Figure 6b. It reveals that the spacing is 0.21 nm for one crystal face (*R*_1_) and 0.24 nm for the neighboring face (*R*_2_). The angle (*θ*) of the two selected crystal faces is 27.94°. From the powder diffraction file (PDF) card 75–1865 for Al_2_O_3_, the interplanar spacing for faces of (113) and (110) is respectively 0.2087 nm and 0.2382 nm, which correspond well with the measured R_1_ and R_2_ in Figure 6b. The angle between the two crystal faces of the hexagonal crystal could be also calculated from Equation (2):

(2) $$\cos\theta =\frac{h_{1}h_{2}+k_{1}k_{2}+\frac{1}{2}(h_{1}k_{2}+h_{2}k_{1})+\frac{3a^{2}}{4c^{2}}l_{1}l_{2}}{\sqrt{\left(h_{1}^{2}+k_{1}^{2}+h_{1}k_{1}+\frac{3a^{2}}{4c^{2}}l_{1}^{2})(h_{2}^{2}+k_{2}^{2}+h_{2}k_{2}+\frac{3a^{2}}{4c^{2}}l_{2}^{2})\right)}}$$

where *h*, *k*, and *l* are defined as the lattice planes.

According to Equation (2), the angle of Al_2_O_3_ between the crystal face (113) and (110) is worked out to be 28°, consistent with the measured *θ* value in Figure 6b. So, the TEM analysis result confirms that the whiskers take on the Al_2_O_3_ phases. Furthermore, it can be seen in Figure 6b that the zone axis of Al_2_O_3_ whiskers is [110], the diffraction spots along the whisker’s axis is the crystal face (003), and its growth direction is parallel to [001], namely, along the *c*-axis. This analysis is in agreement with the calculation results of the surface energy, shown in Figure 2 and Figure 3. The HRTEM image of area 1 is shown in Figure 6c, which further proves that the Al_2_O_3_ whisker is a single crystal. The measured two lattice fringe spacing are 0.43 nm and 0.24 nm respectively, which are well matched with the (003) and (110) planes of the Al_2_O_3_ phases.

### 3.4. The Generation Mechanism for Al_2_O_3_ Whiskers at High Temperatures

**Figure 7:**
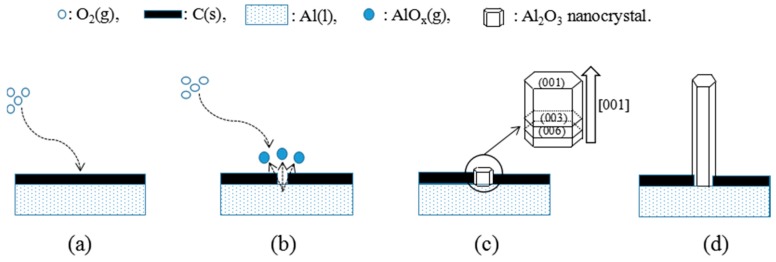
The generation mechanism of Al_2_O_3_ whiskers: (**a**) deposition of O_2_ on molten Al layer; (**b**) generation of AlO_x_(g); (**c**) growth on the direction of [001] and (**d**) the formation of Al_2_O_3_ whiskers.

The generation mechanism of Al_2_O_3_ whiskers at 1300 °C is investigated and simulated in Figure 7. The crystal morphology is determined using different crystal facet growth rates, which could be retarded or accelerated by such factors as impurities, intentionally introduced morphology modifiers, or temperature. In this experiment, the first step involves the melting of metallic Al powders and covering with a layer of graphite at 1300 °C, as shown in Figure 7a. When the oxygen comes in contact with the graphite, the relevant reaction is given by:

Al(l) + C(s) + O_2_(g) →AlO_x_(v) + CO(g) (3)

Equation (3) takes place on the interface of graphite-Al powders. The sub-oxide AlO_x_ in the vapor state will continuously appear with the proceeding of the above reaction. Articles [23,24] also reported that the sub-oxide AlO_x_ in the carbon thermal reaction could be generated. Then, the sub-oxide AlO_x_ is oxidized to Al_2_O_3_ by oxygen in the Ar atmosphere, which forms Al_2_O_3_ nanocrystalline in the surface of the base crystal (shown in Figure 7c).

The surface energy’s effect on the growth morphology is essential to control the growth and atoms state, as shown in Figure 2 and Figure 3. According to the above surface energy calculation results, determining that the (001) surface has the higher surface energy, the driving force for crystal growth would be more intense in the (001) plane, and the alumina crystal would tend to grow preferentially along the direction of the (001) plane from the tip of the crystal (shown in Figure 7c). As a result, the crystal sections of Al_2_O_3_ whiskers turn into the shape of whiskers with [001] orientation in hexagonal crystals, namely along the *c*-axis (shown in Figure 7d). These results agree with the experimental results in Figure 6. The growth mechanism of Al_2_O_3_ whiskers could be followed by the vapor–solid (VS) mechanism.

## 4. Conclusions

(1) Density of States calculation of Al_2_O_3_ showed that Al-O bonding exhibited strong ionic characteristics and the direct band gap between the top of the valence band and the bottom of the conduction band is 6.704 eV. The surfaces of (001), (110), and (113) would be terminated and determined by the Al or O layer.
(2) Under oxygen-rich conditions, the surface energies of low Miller index surfaces of Al_2_O_3_ followed the sequence of (001)-Al > (110)-Al > (113)-Al > (113)-O > (001)-O > (110)-O. Under oxygen-deficient conditions, the sequence was converted to the trend of (001)-O > (110)-O > (113)-O > (001)-Al > (113)-Al > (110)-Al. The (001) surface possessed the higher surface energy.
(3) The growth experiment of Al_2_O_3_ whiskers showed that the whiskers were 50–500 nm in diameter and hundreds of microns in length. The diffraction spots along the alumina whiskers’ axis was the crystal face (003), and its growth direction was parallel to [001], namely, along the *c*-axis. This analysis was in agreement with the calculation and modeling results based on DOS.

## Figures and Tables

**Table 1:** The calculated lattice parameters and theoretical values.

Sources	a (Å)	α (°)
Calculated	5.12	55.28
Reference [12]	5.06	55.31
Reference [13]	5.08	55.30
Reference [14]	5.14	55.17
